# Supplementary material for: Central pattern generator control of a vertebrate ultradian sleep rhythm
Source: Nature. 2024 Nov 6;636(8043):681–9. doi: 10.1038/s41586-024-08162-w (PMC11655359; doi:10.1038/s41586-024-08162-w)
Supplement: Supplementary file 1 — Reporting Summary [file 41586_2024_8162_MOESM1_ESM.pdf]

nature portfolio

Corresponding author(s): Lorenz Fenk
Gilles Laurent

Last updated by author(s): Sep 12, 2024

Reporting Summary

Nature Portfolio wishes to improve the reproducibility of the work that we publish. This form provides structure for consistency and transparency in reporting. For further information on Nature Portfolio policies, see our Editorial Policies and the Editorial Policy Checklist.

Statistics

For all statistical analyses, confirm that the following items are present in the figure legend, table legend, main text, or Methods section.

n/a	Confirmed	
☐	☒	The exact sample size (*n*) for each experimental group/condition, given as a discrete number and unit of measurement
☐	☒	A statement on whether measurements were taken from distinct samples or whether the same sample was measured repeatedly
☐	☒	The statistical test(s) used AND whether they are one- or two-sided<br>*Only common tests should be described solely by name; describe more complex techniques in the Methods section.*
☒	☐	A description of all covariates tested
☒	☐	A description of any assumptions or corrections, such as tests of normality and adjustment for multiple comparisons
☐	☒	A full description of the statistical parameters including central tendency (e.g. means) or other basic estimates (e.g. regression coefficient) AND variation (e.g. standard deviation) or associated estimates of uncertainty (e.g. confidence intervals)
☐	☒	For null hypothesis testing, the test statistic (e.g. *F*, *t*, *r*) with confidence intervals, effect sizes, degrees of freedom and *P* value noted<br>*Give P values as exact values whenever suitable.*
☒	☐	For Bayesian analysis, information on the choice of priors and Markov chain Monte Carlo settings
☒	☐	For hierarchical and complex designs, identification of the appropriate level for tests and full reporting of outcomes
☐	☒	Estimates of effect sizes (e.g. Cohen's *d*, Pearson's *r*), indicating how they were calculated

Our web collection on statistics for biologists contains articles on many of the points above.

Software and code

Policy information about availability of computer code

Data collection	Cheetah (Neuralynx) for recordings using 32-channel CambridgeNeurotech and NeuroNexus probes (version 6.4.2).<br>SpikeGLX for Neuropixels recordings (v.20200309);.<br>Zen 2.1 and 3.1 (Carl Zeiss) was used for image acquisition.
Data analysis	Python (version 3.11.5) and MATLAB (MathWorks) version R2021a and R2018a.<br>Python packages used: scipy (1.11.4), numpy (1.24.3), pandas (2.0.3), and xarray (2023.6.0).<br>Code available at: https://brain.mpg.de/research/laurent-department/software-techniques<br>DeepLabCut (version 2.3.0).<br>Ironclust (4.2.3) was used for spike sorting and manual curation.

For manuscripts utilizing custom algorithms or software that are central to the research but not yet described in published literature, software must be made available to editors and reviewers. We strongly encourage code deposition in a community repository (e.g. GitHub). See the Nature Portfolio guidelines for submitting code & software for further information.

Data

Policy information about availability of data

All manuscripts must include a data availability statement. This statement should provide the following information, where applicable:

- Accession codes, unique identifiers, or web links for publicly available datasets
- A description of any restrictions on data availability
- For clinical datasets or third party data, please ensure that the statement adheres to our policy

Data will be available upon reasonable request.

Research involving human participants, their data, or biological material

Policy information about studies with human participants or human data. See also policy information about sex, gender (identity/presentation), and sexual orientation and race, ethnicity and racism.

Reporting on sex and gender	not applicable
Reporting on race, ethnicity, or other socially relevant groupings	not applicable
Population characteristics	not applicable
Recruitment	not applicable
Ethics oversight	not applicable

Note that full information on the approval of the study protocol must also be provided in the manuscript.

Field-specific reporting

Please select the one below that is the best fit for your research. If you are not sure, read the appropriate sections before making your selection.

☒ Life sciences ☐ Behavioural & social sciences ☐ Ecological, evolutionary & environmental sciences

For a reference copy of the document with all sections, see nature.com/documents/nr-reporting-summary-flat.pdf

Life sciences study design

All studies must disclose on these points even when the disclosure is negative.

Sample size	No statistical tests were used to predetermine sample sizes. We established that our sample sizes are sufficient based on previous experience and commonly used sample sizes in this field of research, taking into account the unusual nature and limited availability of the animal species studied.
Data exclusions	Experiments with off-target placement of electrodes, or tracer injections were excluded from our analysis.
Replication	Main results contain multiple data sets in which findings could be replicated (see exact n-numbers in Figures and their legends)
Randomization	Animals were not assigned to groups, and were selected based on weight and healthy appearance. Randomization was not relevant for our study.
Blinding	Investigators were not blinded to group allocation during data collection and analysis. Our study was mostly observational in nature, measurements were fully automated, and blinding was thus not relevant for our study.

Reporting for specific materials, systems and methods

We require information from authors about some types of materials, experimental systems and methods used in many studies. Here, indicate whether each material, system or method listed is relevant to your study. If you are not sure if a list item applies to your research, read the appropriate section before selecting a response.

Materials & experimental systems

n/a	Involved in the study
☒	☐ Antibodies
☒	☐ Eukaryotic cell lines
☒	☐ Palaeontology and archaeology
☐	☒ Animals and other organisms
☒	☐ Clinical data
☒	☐ Dual use research of concern
☒	☐ Plants

Methods

n/a	Involved in the study
☒	☐ ChIP-seq
☒	☐ Flow cytometry
☒	☐ MRI-based neuroimaging

Animals and other research organisms

Policy information about studies involving animals; ARRIVE guidelines recommended for reporting animal research, and Sex and Gender in Research

Laboratory animals	Adult Lizards (Pogona vitticeps), weighing 150-350g (age ~1.5 to 3years) bred and housed in our state-of-the-art animal facility.
Wild animals	This study didn't involve wild animals.
Reporting on sex	Our findings apply to either sex, which has been assigned based on visual and/or ultrasound inspection. Experimental animals have been chosen based on availability, weight and healthy appearance, irrespective of their sex. We did not perform any sex-based analysis, but our results are highly consistent across all animals studied.
Field-collected samples	This study did not involve field-collected samples.
Ethics oversight	All experimental procedures were approved by the relevant animal welfare authority (Regierungspräsidium Darmstadt, Germany) and conducted following the strict federal guidelines for the use and care of laboratory animals (permit numbers V54-19c20/15-F126/1005_1011 and 2006).

Note that full information on the approval of the study protocol must also be provided in the manuscript.

Plants

Seed stocks	NA
Novel plant genotypes	NA
Authentication	NA
